# Psychosocial reintegration post-traumatic spinal cord injury in Rwanda: An exploratory study

**DOI:** 10.4102/sajp.v80i1.1996

**Published:** 2024-02-29

**Authors:** Maurice Kanyoni, Lena N. Wikmar, Joliana Philips, David K. Tumusiime

**Affiliations:** 1 Department of Physiotherapy, School of Health Sciences, University of Rwanda, Kigali, Rwanda; 2 Department of Neurobiology, Care Sciences and Society, Division of Physiotherapy, Karolinska Institutet, Stockholm, Sweden; 3 Department of Physiotherapy, School of Health Science, University of the Western Cape, Cape Town, South Africa

**Keywords:** community reintegration, spinal cord injury, Rwanda, paraplegia, quadriplegia, resource-constrained, East Africa

## Abstract

**Background:**

Traumatic spinal cord injury (TSCI) survivors are confronted by both physical and psychosocial barriers when returning to their communities. Therefore, reintegration is an important aspect of their journey back into social life.

**Objectives:**

To assess psychosocial reintegration after TSCI in Rwanda.

**Method:**

All community-dwelling adults who were registered in the previous epidemiological study were recruited and injury characteristics questionnaire and the Sydney Psychosocial Reintegration Scale version 2 (SPRS-2) were used to collect data through a telephone interview.

**Results:**

The study traced 58 participants, 77.6% (*n* = 45) were male and 56.9% (*n* = 33) were categorised with paraplegia. Overall, the results show poor community reintegration. The SPRS-2 and domain mean (SD) scores were: overall SPRS-2 of 20.95 (11.56), occupational activity (OA) of 3.68 (4.31), interpersonal relationship (IR) of 7.11(4.31) and living skills (LS) of 7.43 (5.32). Gender significantly influenced overall SPRS-2 (*p* = 0.011) and two domains: OA (*p* = 0.005) and LS (*p* = 0.012). Level of injury was significantly associated with an OA domain score of SPRS-2 (*p* = 0.002). Gender explained 29% of the variance in the LS domain of SPRS-2, with males reporting better psychosocial reintegration.

**Conclusion:**

Gender strongly predicted psychosocial reintegration following a TSCI, which is an indication of the role of social support.

**Clinical Implications:**

Traumatic SCI rehabilitation should be holistic to help prepare the person to return to the community. There should be an assessment of an individual’s readiness to return to the community before discharge from the hospital.

## Introduction

The incidence of spinal cord injury (SCI) varies widely between countries. The global annual estimate is 15–40 per million population with an average of 23 per million people (Wyndaele & Wyndaele 2006). There are several studies conducted in Africa to highlight the magnitude of traumatic spinal cord injury (TSCI) in Africa. The occurrence rates differ from one country to another. A recent South African study reported an incidence of 75.6 per million people (Joseph et al. 2015). In Botswana, a study reported an incidence of 13 per million people (Löfvenmark et al. 2015) while in Tanzania it was 26 per million people (Moshi, Sundelin & Sörlin 2017). A systematic review conducted in the Middle East and North Africa (MENA) countries showed that the regional incidence is 23.24 per million people (Elshahidi et al. 2018). There are other hospital-based studies in Africa like Ethiopia, Malawi and Nigeria where despite methodological disparities they try to show the likely magnitude of the problem in the respective countries and their communities (Eaton et al. 2019; Lehre et al. 2015; Olasode 2006).

Reintegration refers to acquiring or resuming age, gender, culture relevant roles and/or statuses including independence in decision making and being a productive member of the society (Dijkers 1998). It is contextual and environmental factors that affect an individuals’ participation and hence affect his or her reintegration after TSCI (Dijkers 1998). Spinal cord injury survivors face both physical, economic, and psychosocial challenges when they return to the community (Sekaran et al. 2010). Therefore, assessing factors affecting TSCI survivors wanting to come back into their community is an important aspect of their journey back into the social life.

Health sector in high-income countries has seen improvement and this has brought improved outcomes for individuals with SCI; the reality is very different in the many low-income counterparts (Burns & Connell 2012). Managing acute SCI, providing comprehensive rehabilitation, and ensuring adequate support to allow community reintegration requires adequate resources.

Shortage of wheelchairs has been reported in low-income countries and where they are available are rarely constructed to suit the inaccessible environment (Löfvenmark et al. 2017). Bladder dysfunction management techniques such as clean intermittent catheterisation decrease complication rates compared with indwelling catheters and is recommended for the neurogenic bladder management (Madasa et al. 2020). However, indwelling catheters are commonly used in low-income countries because of lack of catheters and staff to assist with catheterisation and unwilling patients to self-catheterise (Burns & O’Connell 2012; Levy et al. 1998). Bladder and bowel challenges coupled with community attitude, physical environment, unfriendly transport means and access to health facilities complicate reintegration following SCI (Levy et al. 1998).

Rwanda is a mountainous land-locked country situated in East Central Africa. Despite the country being predominantly a farming population, Rwanda has seen significant economic and health sector development in the recent years. In Rwanda, the only available housing arrangement is a family household. Until 2019, there were 11 disability-friendly public transport buses in the country (Republic of Rwanda – Ministry of Infrastructure 2018). There are no SCI-specialised rehabilitation services available in the country. Formal health insurance schemes for the self-employed and rural farmers are difficult to institute for a number of reasons including unpredictable incomes and informal nature of their work. Community Based Health Insurance Schemes (CBHISs) are promising alternatives for a cost-sharing health care system, which hopefully also leads to better utilisation of health care services, reduces illness-related income shocks and eventually leads to a sustainable and fully functioning universal health care system.

Rwanda’s national CBHI scheme (commonly known as ‘mutuelles de santé’) is now one of the largest public health insurance schemes in sub-Saharan Africa with more than 95% coverage of the informal sector (Collins, Saya & Kunda 2016). Rwanda’s population working in the formal sector is covered by the national health insurance and membership is compulsory. However, the CBHI has limitations in relation to rehabilitation services; concerning long-term rehabilitation conditions like SCI and stroke (International Labour Organization 2016). Rehabilitation sessions per month are limited and rehabilitation technologies are not either paid for or paid for only to designated rehabilitation workshops. These coupled with long distances from households to the rehabilitation centres, poor infrastructure to mention a few are likely to limit access to rehabilitation by SCI persons. There are no studies to date that assessed community reintegration of TSCI people and influencing factors in Rwanda. Therefore, there is no solid foundation knowledge on what and how psychosocial reintegration is perceived and influenced. Thus, the aim of this paper is to assess the levels of community reintegration following TSCI in Rwanda.

## Methods and materials

The community reintegration was a follow-up study as part of an initial epidemiological study that registered new TSCI individuals for a period of 1 year from 10th October 2019 to 9th October 2020, in which participants were followed up one year later. This study created a registry in which at least two telephone contacts were included in the retained data for the participant. Data were collected from May 2021 till October 2021; this is a period immediately after the coronavirus disease 2019 (COVID-19) pandemic. The country situation in relation to the pandemic had stabilised and people were moving within the country freely without restrictions. Data were collected using the Sydney Psychosocial Reintegration Scale version-2 (SPRS-2) which was a standard questionnaire (Nizeyimana, Phillips & Joseph 2022; Tate 2011).

## Research setting

The study was conducted in Rwanda, one of the smallest countries on the African continent. The 2022 national census indicated the population of Rwanda as 13 246 394 people (National Institute of Statistics of Rwanda 2022). In Rwanda, the only available housing arrangement is a family household; therefore, all participants were spread all over the country in their respective households in towns and village areas.

## Data-collection procedure and instrumentation

Data were collected via a telephone interview with two questionnaires: (1) Demographic and injury characteristic questionnaire and (2) SPRS-2. The demographic and injury characteristics questionnaire consisted of five items designed to collect demographic and specific injury characteristics like age, gender, marital status, injury level, and cause of injury. The SPRS-2 was first made to asses psychosocial reintegration after traumatic brain injury (TBI) aiming at three domains: (1) occupational activity (OA), e.g. ‘how do you rate your work/study skills’ and ‘how do you rate the number or type of leisure activities or interests’; (2) interpersonal relationships (IR) for example ‘how do you rate your relationship with other family members’ and ‘if you have a spouse/partner, how do you rate your relationship’; (3) living skills (LS), for example ‘how do you rate your living situation’ and ‘how do you rate your social skills’ (Tate 2011). Scores range from 0 to –16 for each domain and 0 to 48 for SPRS-2 overall score. For domain and overall scores, a higher score indicates better psychosocial reintegration. Sydney Psychosocial Reintegration Scale version 2 was initially designed to assess psychosocial reintegration after TBI. Its reliability, validity and sensitivity to change in people with SCI were proved in comparison with other instruments that measure the same outcome; for example the Craig Handicap Assessment and Reporting Technique (CHART), (De Wolf et al. 2010). It was identified to have greater sensitivity to change with overall less ceiling effects compared to CHART (De Wolf et al. 2010). The same study showed that internal consistency is excellent; Cronbach alpha = 0.93, with this therefore, SPRS-2 proves to be a reliable and easy to administer questionnaire.

Data were collected through telephone interview because of the fact that study participants were spread throughout the country making it not feasible to physically meet each of them.

The inclusion criteria were: (1) traceable during follow-up through retained telephone contacts; (2) survival at the time of data collection and (3) a resident of Rwanda at the time of data collection.

The data-collection tool was uploaded as an electronic document and was administered by telephone call. Responses were automatically relayed to an online server downloadable either as an Excel sheet or pdf.

## Data analysis

Study participants’ profile details such as age, gender and injury characteristics were analysed descriptively using IBM Statistical Package for Social Sciences (SPSS version 26, IBM, SPSS, New York, USA). A test for equality of variance (homogeneity) was conducted; the Brown–Forsythe test was used to test the robustness of data. A normality test was conducted to check on distribution of sample and a normal distribution was obtained. *P*-value was set at < 0.05 for all tests including turkey post-hoc test. Descriptive data are presented in the form of frequency and percentages. To assess the level of community reintegration of the participants, mean and standard deviation of scores of individual domains (OA, IR, and LS) and the overall total SPRS-2 score were calculated.

Bivariate linear regression analysis; T-tests; and one-way analysis of variance were used. For groups that showed a significant effect, post-hoc comparison using Turkey’s test was used in order to compare means within-group and between groups. Means of overall SPRS-2 and its domains with demographic and injury variables were calculated. Demographic and injury characteristics that were significantly associated with psychosocial reintegration in bivariate analysis were included in multiple linear regression analyses. Dummy variables were created for all categorical independent variables prior to multiple regression analysis, and several reference group categories were selected and indicated as constants. The final analysis included the development of a regression model that predicted which demographic and injury characteristics affect each domain of psychosocial reintegration.

### Ethical considerations

Ethical clearance to conduct this study, and the study protocol, was obtained and approved by the Institutional Review Board of the College of Medicine and Health Sciences at the University of Rwanda (No. 308/CMHS IRB/2019). The objectives of the study were explained to the participants, participation was voluntary and anybody was free to withdraw at any point of the study without giving reasons. Those who accepted were requested to sign a consent form. All participants consented for follow-up in the following year during discharge at the hospital (during epidemiology study) and were contacted at the time of data collection in the community reintegration study (current study) to voluntarily give a verbal consent. Codes were used instead of names, participant demographic, injury and reported information was kept in a computer software accessible only by the first author. Authors declare that they complied with the Helsinki recommendations and the Ministry of Health, Republic of Rwanda guidelines on human research participants.

## Results

### Overview of participants

**FIGURE 1:**
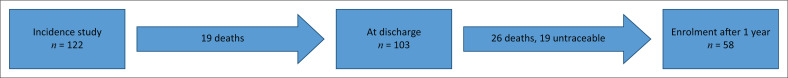
Participant enrolment after 1-year post discharge.

The incidence study registered 122 participants who sustained a TSCI and out of them 19 died in acute care and 103 survived till discharge. Participants were followed up after 1-year post-discharge; 26 participants had died and 19 were untraceable. The main reasons for failure to trace the participants in the community were provided contact telephones not on air for 3 weeks, not picking calls for 3 weeks and provided telephone number in the acute care was not his or hers and the telephone owner no longer lives with the TSCI individual. Through provided telephone contacts or during the community tracing exercise, we identified 58 participants with TSCI (Figure 1).

### Participants’ injury characteristics

This study managed to trace 58 participants in the community. All of them consented to take part in the study. Slightly more than 77% (77.6%, *n* = 45) were male, and more than half (56.9%, *n* = 33) were categorised as paraplegia. One third (32.8%, *n* = 19) of the participants were in the youthful age bracket of 18–33 years. The most common cause of TSCI was falls (74.1%, *n* = 43) followed by road traffic accidents (15.5%, *n* = 9). Table 1 shows the general characteristics of study participants. The SPRS-2 and domains mean (SD) scores were: overall SPRS-2 of 20.95 (11.56), occupational activity of 3.68 (4.31), Interpersonal relationships of 7.11 (3.85) and living skills of 7.43 (5.32). The overall SPRS-S score was low indicating poor community reintegration of the participants.

**TABLE 1:** General characteristics of the study sample (*N* = 58).

Variable	Frequency (*n*)	%
**Age group (years)**		
18–33	19	32.8
34–49	18	31.0
50–65	17	29.3
66 and above	4	6.9
**Gender**		
Male	45	77.6
Female	13	22.4
**Marital status**		
Married	29	50.0
Unmarried	21	36.2
Widowed	8	13.8
**Cause of injury**		
Sports	1	1.7
Assault	2	3.4
Transport	9	15.5
Fall	43	74.1
Other causes	3	5.2
**Level of injury**		
Paraplegia	33	56.9
Tetraplegia	25	43.1
**Severity of injury**		
Complete lesion	18	31.0
Incomplete lesion	40	69.0
**Paraplegia and ASIA classification**		
A	14	42.4
B	-	-
C	10	30.3
D	9	27.3
**Tetraplegia and ASIA classification**		
A	13	52.0
B	-	-
C	4	16.0
D	8	32.0
**Occupation or works before injury**		
Farming or subsistence	22	37.9
Subsistence and excess for sell	11	19.0
Business	4	6.9
Monthly or formal sector salary	13	22.4
Wage	5	8.6
Not working	3	5.2
**Occupation or works after injury**		
Farming or subsistence	6	10.3
Subsistence and excess for sell	4	6.9
Business	2	3.4
Monthly or formal sector salary	9	15.5
Wage	3	5.2
Not working	34	58.6

ASIA, American spinal cord injury association.

### Association between demographic and injury characteristics and Sydney Psychosocial Reintegration Scale version 2 and its domains

The comparison between demographic and injury characteristics and overall SPRS-2 and its domains (OA, IR, LS) were assessed through bivariate linear regression analysis, and the results are shown in Table 2. Two independent variables: one demographic and the other injury variable, were significantly associated with the overall mean SPRS-2 scores and its two domains. Gender was significantly associated with overall SPRS-2 (*p* = 0.011), OA (*p* = 0.005) and LS (*p* = 0.012) while level of injury was significantly associated with OA domain score (*p* = 0.002). There was no significant association between age, marital status and severity of injury and SPRS-2 and all its domains.

**TABLE 2:** Linear regression analysis results of overall Sydney psychosocial reintegration scale version 2, individual domain scores with general characteristics of the study sample.

Variable	SPRS-2			OA			IR			LS		
	Mean	SD	*p*	Mean	SD	*p*	Mean	SD	*p*	Mean	SD	*p*
**Age group (years)**	-	-	1.000	-	-	0.971	-	-	0.865	-	-	0.985
18–33	20.95	13.0	-	3.47	4.3	-	7.00	3.9	-	7.50	5.9	-
34–49	20.83	11.7	-	4.06	5.3	-	7.28	4.1	-	7.12	5.8	-
50–65	21.18	11.1	-	3.44	3.7	-	6.69	3.3	-	7.76	4.9	-
66 and above	20.50	9.9	-	4.00	2.9	-	8.50	5.8	-	7.00	4.1	-
**Gender**	-	-	0.011*	-	-	0.005*	-	-	0.310	-	-	0.012*
Male	22.29	12.2	-	4.14	4.7	-	7.37	4.0	-	8.23	5.5	-
Female	16.31	7.6	-	2.15	2.0	-	6.23	3.3	-	4.50	3.4	-
**Marital status**	-	-	0.806	-	-	0.909	-	-	0.461			0.949
Married	21.69	12.0	-	3.93	5.0	-	7.71	3.8	-	7.46	5.6	-
Unmarried	20.81	11.7	-	3.48	3.8	-	6.70	3.7	-	7.60	5.4	-
Widowed	18.63	10.7	-	3.29	3.1	-	6.00	4.5	-	6.88	4.6	-
**Level of injury**	-	-	0.194	-	-	0.002*	-	-	0.280	-	-	0.615
Paraplegia	21.18	12.5	-	3.72	4.1	-	7.06	4.0	-	7.34	5.5	-
Tetraplegia	20.64	10.5	-	3.63	4.6	-	7.17	3.6	-	7.54	5.2	-
**Severity**	-	-	0.764	-	-	0.644	-	-	0.828	-	-	0.672
A	20.26	11.2	-	3.19	3.8	-	6.81	3.8	-	6.85	5.1	-
C	20.14	11.9	-	3.62	4.9	-	7.62	4.4	-	7.43	6.2	-
D	22.71	12.4	-	4.47	4.7	-	7.18	3.5	-	8.38	5.0	-

Note: Values are presented as means and standard deviations of overall SPRS-2 and its three domains compared using bivariate linear regression model.

SPRS-2; Sydney psychosocial reintegration scale version 2, OA, occupation activities, IR, interpersonal relationships; LS, living skills.

* , significant values.

### Multivariate regression analysis

Bivariate regression analysis resulted in two independent variables that are significantly influencing SPRS-2 and its domains (OA and LS) at *p* value of less than 0.05. In order to obtain which independent variables actually predict the outcome variable, the multiple regression model was computed to determine independent predictors of overall SPRS-2 and its two separate domains (OA and LS). Results of the model are presented in Table 3.

**TABLE 3:** Multivariate regression analysis results for overall Sydney Psychosocial Reintegration Scale version 2 and individual domain scores, gender and level of injury.

Variable	Group	SPRS-2		OA		LS	
		Standardised β coefficient	*p*	Standardised β coefficient	*p*	Standardised β coefficient	*p*
Gender	Male	0.218	0.101	0.196	0.147	0.290	0.030*
	Female (constant)	-	-	-	-	-	
Level of injury	Paraplegia	-	-	0.011	0.937	-	-
	Tetraplegia (constant)	-	-	-	-	-	-

Note: Multivariate regression model showing independent variables that influence overall SPRS-2 and its domains.

SPRS-2; Sydney psychosocial reintegration scale version 2, OA, occupation activities; LS, living skills.

* , significant values.

Gender explained 29% of the variance in the LS domain of SPRS-2 (standardised coefficient = 0.290, *p* = 0.030), with males reporting better psychosocial reintegration compared to females. In the occupational activity domain, level of injury did not predict reintegration in the sample; *p* > 0.05.

## Discussion

The main objective of this study was to assess psychosocial reintegration after TSCI in Rwanda. The study results show poor community reintegration of TSCI people but also showed that gender and level of injury significantly influenced community reintegration possibilities of the study participants. The study participants were dominated by gender disparity with male participants being more than females, which is a general pattern even in other studies conducted in other communities elsewhere (Forchheimer, Kalpakjian & Tate 2004; Scovil et al. 2012; Sekaran et al. 2010). The observed gender difference at the initial identification study (122 participants) and later enrolment (current study) might be because of the fact that in the African context males are more active in the work force both in formal and informal sectors and are also more mobile. Therefore, there is likelihood of male sustaining TSCI because of work-related causes and road traffic accidents. However, there is a need to look at other reasons other than gender proportions such as the prevalence of complications. A recent study reported a higher prevalence of complications immediately after injury and females are more affected than males (Raguindin, Muka & Glisic 2021). This might lead to female mortality in acute care and immediately after discharge. The epidemiology study registered acutely injured TSCI (122 participants) and reported in-hospital mortality of 14.75%, which mostly included females This might explain the observed gender disparity in the follow-up period.

Gender was seen to significantly influence community reintegration with males more being better prepared to resume normal roles and responsibilities in the community compared to females. Similar results were reported by Forchheimer et al. (2004), and Buys et al. (2022) in which women experienced more environmental barriers; hence, poor community reintegration. This gender skewness in community reintegration in the African context might be because of unemployment among women with TSCI but also unmet community needs of women.

The meaning of community integration differs within the literature, but there are common areas that include relationships with others, independence in activities of daily living and meaningful occupation (De Wolf et al. 2010). A definition that is relevant to the SCI population would be; ‘community reintegration is resuming age/gender/and culturally appropriate roles/statuses/activities, including independence/interdependence in decision making, and productive behaviours performed as part of multivaried relationships with family, friends, and others in natural community settings’ (Sekaran et al. 2010). Participation is another aspect that is also closely related with community reintegration and is defined as taking part in life situations (World Health Organization 2001). The results of this study show that the overall mean score of community reintegration is low, suggesting that the sample is faced with poor community integration. This is consistent with results of studies conducted elsewhere in Africa, Nepal, and India (Scovil et al. 2012; Sekaran et al. 2010; Gretschel, Visagie & Inglis 2017; Nizeyimana et al. 2020). Low scores of SPRS-2 that translate into poor community reintegration reported in this study suggest possible challenges and barriers to community reintegration. In most African countries, individuals rely on their physical abilities to cater for themselves and their families, most of the time through manual labour such as farming. A physical disability greatly reduces one’s survival advantage. Poor community reintegration reported in the current study might be because of physical and cultural factors and shortage of assistive technology common in low-resource settings as reported in a review by Burns and Connell (2012).

The level of injury greatly affects function after injury and in turn this is likely to impact on ones’ ability to return to his or her normal roles and function. This study showed that the level of injury is a predictor of community reintegration in which people have low level of injury; paraplegia is likely to be better reintegrated than those with high injuries; tetraplegia. This is also reported in other studies (Atobatele, Olaleye & Adebisi 2018; Olckers 2017). This is partly because of more functional impairments, activity limitation and community participation restrictions that a high-level SCI person is likely to face compared to a low-level SCI injury person. However, another study conducted in South Africa reported a different pattern in which level of injury does not influence community reintegration (Nizeyimana et al. 2022). This disparity between the results of this study and the South African study might be because of access to rehabilitation services, government support and the time from discharge to the data collection.

Spinal cord injury people in low-resource settings are faced with different challenges in their community like harsh terrains, inaccessible homes and communities and physical altitude. These factors are exacerbated by societal attitude towards people with severe physical disabilities that a TSCI survivor is likely to face depending on the severity (Burns & Connell 2012). Rehabilitation ideally should aim at full inclusion and participation of people with disabilities in the physical and psychosocial environment. However, most of what occurs in SCI rehabilitation is directed towards reducing or at most minimising functional limitations (Scelza et al. 2007). Specific intervention programs to maximise community integration are limited as a result; the potential for full reintegration of a person with SCI into the community is unmet in poor resource settings.

The current study did not find any significant relationship between demographic variables such as age, marital status and injury characteristics like injury severity and community reintegration.

Age was analysed as categorical data recommended by the Executive Committee for the International SCI Data Sets. This is consistent with findings by Scelza et al. (2007) in which the author assessed barriers and opportunities for community reintegration for people with SCI and found out that demographic factors are not long-term predictors of community reintegration following SCI.

### Strength

This is the first study to assess community reintegration after SCI in Rwanda. The methodology of the study used international standard tools to collect data, which allows comparison with other data collected from other countries.

### Limitation

The main limitation of this study is a small sample as some participants died or were untraceable in the community; a large sample may yield different results. Another limitation is the fact that the instrument used in data collection is not tested for reliability and validity in the Rwandan context. It has been used in a study to assess psychosocial reintegration among South Africans with TSCI by Nizeyimana et al. (2022), but there is a need to conduct a validation study of SPRS-2 among Rwandans.

## Conclusion

The community reintegration level of TSCI people in this study is generally low. The idea of community reintegration is challenged based on one’s role in the community and this would explain why gender has been identified as a predictor of an individual’s level of community reintegration. The level of injury determines functional ability after an injury; this is an indication that rehabilitation is an important facilitator of community reintegration.
